# Gelation process visualized by aggregation-induced emission fluorogens

**DOI:** 10.1038/ncomms12033

**Published:** 2016-06-23

**Authors:** Zhengke Wang, Jingyi Nie, Wei Qin, Qiaoling Hu, Ben Zhong Tang

**Affiliations:** 1 MOE Key Laboratory of Macromolecular Synthesis and Functionalization, Department of Polymer Science and Engineering, Zhejiang University, Hangzhou 310027, China; 2 Key Laboratory of Adsorption and Separation Materials and Technologies of Zhejiang Province, Hangzhou 310027, China; 3 Department of Chemistry, Hong Kong Branch of Chinese National Engineering Research Center for Tissue Restoration and Reconstruction, Hong Kong University of Science and Technology, Clear Water Bay, Hong Kong 999077, China

## Abstract

Alkaline-urea aqueous solvent system provides a novel and important approach for the utilization of polysaccharide. As one of the most important polysaccharide, chitosan can be well dissolved in this solvent system, and the resultant hydrogel material possesses unique and excellent properties. Thus the sound understanding of the gelation process is fundamentally important. However, current study of the gelation process is still limited due to the absence of direct observation and the lack of attention on the entire process. Here we show the entire gelation process of chitosan LiOH-urea aqueous system by aggregation-induced emission fluorescent imaging. Accompanied by other pseudo *in situ* investigations, we propose the mechanism of gelation process, focusing on the formation of junction points including hydrogen bonds and crystalline.

## Abstract (web summary)

Chitosan hydrogel materials have unique properties, but understanding the gelation process these materials undergo has been problematic. Here, Wang and co-workers have visualised the entire gelation process of a chitosan LiOH-urea aqueous system by aggregation induced emission fluorescent imaging.

## Graphical abstract

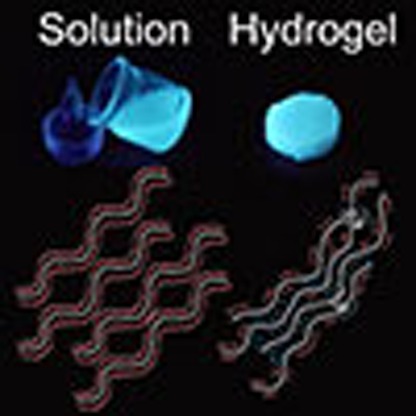

Polysaccharide has gained tremendous attention as a useful and renewable resource^1^. As one of the most important polysaccharide, chitosan (CS) has generated a great deal of interest and provides a wide range of applications^2^,^3^,^4^,^5^,^6^,^7^,^8^. In the utilization of CS material, hydrogel is a major and vital branch^9^. Numerous methods have been proposed for the fabrication of CS based hydrogels. Generally speaking, CS based hydrogel can be fabricated via covalent/ionic cross-linking, complexation with another polymer or aggregation after CS grafting and so on^2^,^10^,^11^. Solubilization of CS in an acidic aqueous medium is the simplest and conventional way to prepare a CS hydrogel. However, the lack of mechanical strength of CS hydrogels via acidic solvent has been a serious impediment. Several methods such as irradiation and reinforcement have been used to enhance their mechanical strength. But these methods are not very desirable due to the sacrifice of intrinsic properties of CS^12^. Fortunately, a class of novel solvent system, alkali-urea aqueous solutions, had been introduced^13^,^14^. The resultant CS hydrogel showed significant improvement in hardness, strength and toughness without any cross-linking agents^13^. Furthermore, the distinct gelation behaviour offers innovative hydrogel design strategy^15^. Thus, the study of gelation mechanism shows great industrial and academic value.

Although hypothesis of gelation mechanism had been discussed, understanding of the gelation process is still limited^12^,^13^,^14^. First, in previous studies only the thermal gelation of CS alkali-urea aqueous solution was in the centre of focus. However, a rinse procedure is required after thermal gelation to remove LiOH and urea. The rinse procedure is not only a prerequisite of any application, but also a crucial stage in the formation of robust CS hydrogel according to our research. Second, although visualization is the most powerful way in the investigation of gelation process, there are several defects in the current studies. First, for observation like scanning electron microscope or transmission electron microscope, the investigation suffers from a disadvantage because the native stage of hydrogel is characterized by the presence of water and the need to remove water before examinations inevitably affects the morphology of the hydrogel^16^. Second, due to the existence of large amount of LiOH and urea, the formation of salt crystals could hardly be avoided, which is interference in the observation. Third, the transformation during gelation process had not been studied. In summary, it is necessary to monitor the CS LiOH-urea system *in situ* and in real time, with the entire gelation process involved.

Fluorescent imaging is an ideal tool for such research purpose. Nevertheless, the research on the gelation process is internally related to the evolution of aggregation state for CS LiOH-urea system^17^. For conventional fluorescent agents, aggregation leads to weakened emission due to the notorious aggregation-caused quenching effect^18^. Opposite to the common aggregation-caused quenching effect, a unique photophysical process termed aggregation-induced emission (AIE) had been observed^19^. The fluorescence of AIE fluorogens is boosted by the restriction of intramolecular rotation^18^. So an AIE fluorogen becomes highly emissive in the aggregation state^20^.

Tetraphenylethene is an archetypical AIE fluorogen. In our previous work, we synthesized a novel AIE fluorogenic probe, that is, tetraphenylethene-labelled chitosan (TPE-CS)^21^,^22^. TPE-CS is a perfect candidate for the investigation on gelation process. Unlike conventional fluorescent agents such as fluorescein isothiocyanate, tetraphenylethene is photostable in the strong alkaline medium^23^. Aggregates formation in the system renders stronger photoluminescence rather than causing quenching. Besides fluorescent images, TPE-CS also provides fluorescent spectroscopy information, which is correlated to the aggregation state of system. In the present work, AIE fluorogens are employed to realize the visualization of the gelation process of CS LiOH-urea solution. The visual characterization is supported by other *in situ* or pseudo *in situ* investigations, focusing on the formation of junction points. Finally, an understanding of gelation mechanism is proposed, covering the unique and intriguing transition from solution to hydrogel.

## Results

### Fluorescent TPE-CS hydrogel via LiOH-urea solvent

**Figure 1:**
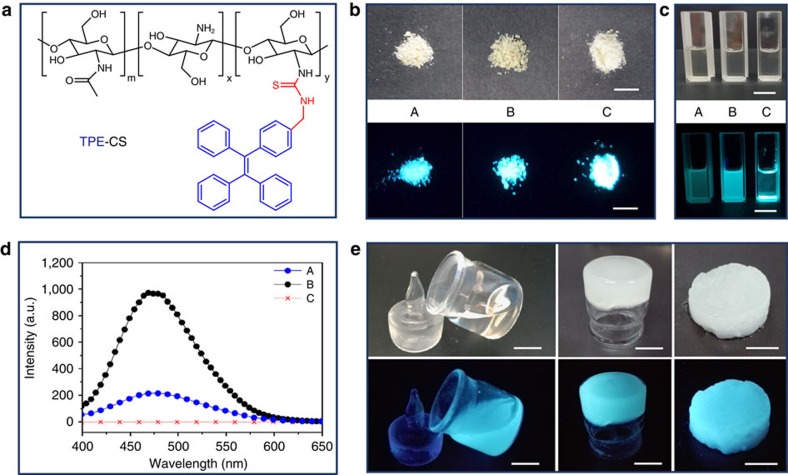
TPE-CS samples in the form of powder solution and hydrogel. (**a**) Molecular structure of TPE-CS. (**b**) Digital images of powder samples; Scale bar, 1.0 cm. (**c**) TPE-CS samples after treated by freeze-thaw dissolving procedure in LiOH-urea aqueous solution; Scale bar, 1.0 cm. (**d**) Photoluminescence spectra of TPE-CS solution, the intensity corresponding to sample C was denoted as zero, since sample C cannot be dissolved in the LiOH-urea solvent. (**e**) Digital images of formation of hydrogel with TPE-CS: solution of TPE-CS, TPE-CS gel after thermal gelation, and TPE-CS hydrogel after complete removal of LiOH and urea; Scale bar, 5.0 mm. The fluorescent photographs were taken under ultraviolet illumination (365 nm).

Three TPE-CS samples, molecular structure of TPE-CS shown in Fig. 1a, were prepared with different degree of labelling (DL). ^1^H NMR spectra of TPE-CS showed that labelling the CS with tetraphenylethene fluorogens had been successfully done (Supplementary Fig. 1). The DL of CS was 0.93, 1.54 and 3.56 mol% for the three samples respectively. These samples were denoted as A, B and C with the increase of DL. The powders of TPE-CS samples were light yellow in appearance under normal room lighting and emitted a strong blue light on ultraviolet illumination (Fig. 1b). These TPE-CS samples showed different solubility in LiOH-urea aqueous solvent (Fig. 1c). Sample A and B formed transparent solution, while sample C only existed as sediment. The results showed that the solubility of TPE-CS in LiOH-urea aqueous solution was reduced by the increase of DL, owing to the hydrophobic tetraphenylethene fluorogens. However, the fluorescence of the TPE-CS is intensified with the increase of DL, which was shown in the ultraviolet photographs (Fig. 1c) and validated by the photoluminescence (PL) spectra (Fig. 1d).

Thus the DL should be controlled by balancing two opposite factors. On one hand, heavily labelled sample was favoured for strong photoluminescence signal. On the other hand, lower DL was favoured to minimize interference in the behaviour of CS. Taking both factors into consideration, sample B was optimum and was utilized as the raw material in the present work.

Typical preparation procedures of CS hydrogel via LiOH-urea solvent were demonstrated in Supplementary Fig. 2, including thermal gelation and the succedent rinse procedure. TPE-CS can be fabricated into hydrogel by the same preparation procedures (Fig. 1e). In addition, the gelation behaviour of CS and TPE-CS was tested by rheological method (Supplementary Fig. 3)^24^. The well-matched gelation time of CS and TPE-CS indicated that, with proper DL, the introduction of tetraphenylethene fluorogens did not exert much interference on the gelation process of CS.

### Visualization of gelation process

**Figure 2:**
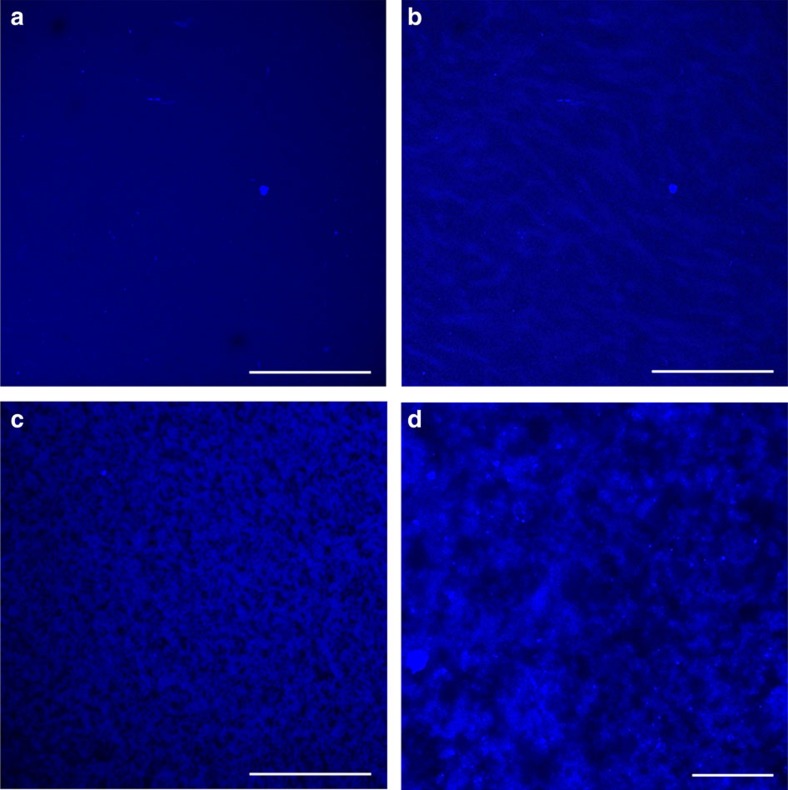
Confocal laser scanning fluorescence microscope images of the gelation process of TPE-CS. (**a**) solution; (**b**) gel after thermal gelation; (**c**,**d**) hydrogel after rinse procedure; Scale bar, 250 μm (**a**–**c**); 25 μm (**d**).

CS gelation process was monitored by confocal laser scanning fluorescence microscope (Fig. 2). In previous studies, extremely dilute CS solution (10^−6^ g ml^−1^) was used for imaging, with drying procedure involved^12^. However, for the preparation of hydrogel with high mechanical performance, the concentration of CS was in the range of 10^−2^ g ml^−1^. So the *in situ* visualization within this concentration range would provide more direct and effective information on the gelation process. The fluorescent image of solution showed no specific patterns (Fig. 2a). However, some bright areas started to appear after heat absorption started, corresponding to the thermal gelation stage (Supplementary Fig. 4). As time goes on, the bright areas kept developing. Then the development slowed down, and finally the pattern became stable (Fig. 2b). This indicated that the thermal gelation of the system possessed certain terminal point. However, after LiOH and urea were completely removed the structure of gel further developed (Fig. 2c). This indicated that the terminal point of thermal gelation was not the termination of gelation process. After rinse stage, the bright areas subdivided and contracted, forming dark areas. Eventually, CS hydrogel came into being with a reticular structure (Fig. 2d and Supplementary Fig. 5). In addition to fluorescent images, TPE-CS also provides fluorescent spectroscopy information, which was in good accordance with the aggregation state of system (Supplementary Figs 6,7 and Supplementary Discussion). The results above indicated that gelation initiated after heat absorption, however, the rinse stage was a crucial stage in the formation of CS hydrogel structure.

**Figure 3:**
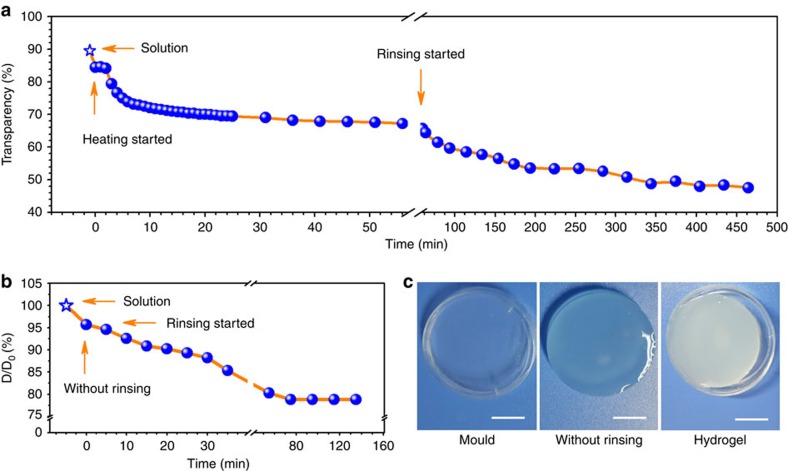
Change of macroscopic properties of CS LiOH-urea system in the gelation process. (**a**) Evolution of transparency of CS LiOH-urea system. (**b**) Dimension shrinkage rate of CS gel, D_0_ is the diameter of mould and D is the diameter of gel. (**c**) Images of CS hydrogel and the mould used in preparation; Scale bar, 1.0 cm.

In addition to the fluorescent study, macroscopic property evolution of CS LiOH-urea system was studied by pseudo *in situ* investigations to further understand the gelation process. Optical transparency reflected the homogeneity of materials. In the gelation process, the solution was transparent originally, however, certain structure arose due to gelation, thus increased the light scattering and decreased the transparency of system^25^. Figure 3a showed the transparency as a function of time in the entire gelation process. First, the transparency maintained for a short period after contacted with heat source. Then the transparency gradually decreased and finally reached a constant value, corresponding to the terminal point of thermal gelation stage. The decrease continued in the rinse stage, indicating the further development of structure in this stage. The shrinking behaviour in the gelation process is an important physical phenomenon of hydrogel, which is closely related to the solvophobic and solvophilic interactions. The shrinking behaviour of CS LiOH-urea system was followed in the entire process (Fig. 3b). The volume of gel slightly decreased after thermal gelation stage. When the gel was immersed in water after thermal gelation, it showed evident shrinkage rather than swelling, and the diameter of CS hydrogel was 78% of the value of mould (Fig. 3c). Moreover, the volume change was not a simple shrinkage caused by the loss of components. In addition, the removal of OH^−^ is closely related to the volume change of CS gel (Supplementary Figs 8,9). The results mentioned above strongly indicated that the shrinkage was due to the further development of gel structure. The evolution of macroscopic property during the gelation process was in good accordance with the information provided by fluorescent images. In addition to thermal gelation, the CS LiOH-urea system went through crucial transformation in the rinse stage.

### Formation and development of junction points

To further understand the formation and development of junction points during the two stages, other *in situ* or pseudo *in situ* investigations were employed.

**Figure 4:**
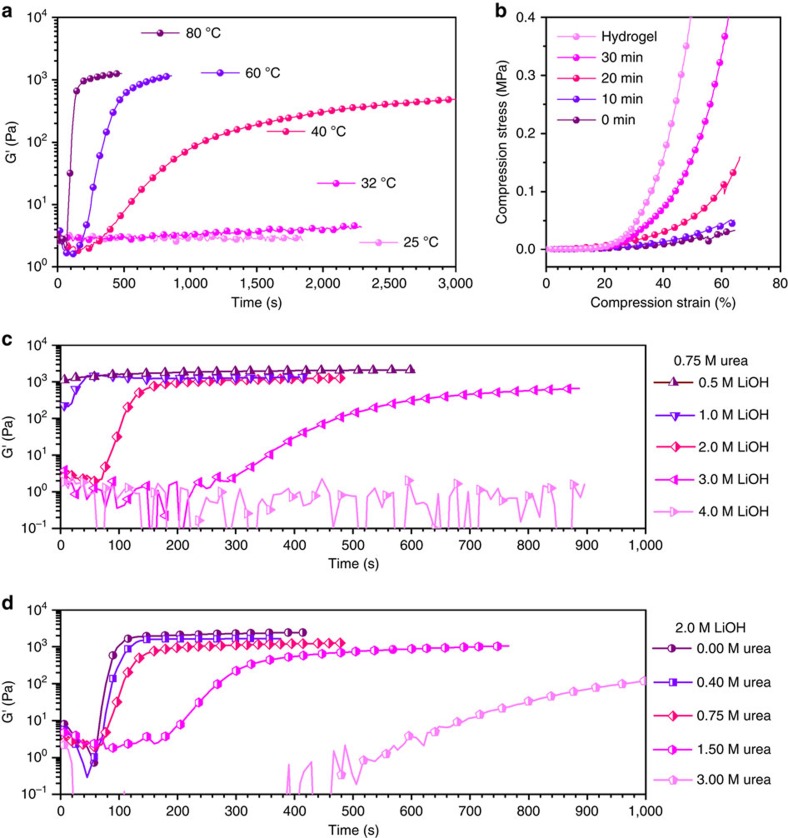
Strength evolution of CS LiOH-urea system in the entire gelation process. (**a**) Evolution of storage modulus (G') of CS LiOH-urea solution with time; the solutions were cured at marked temperatures. (**b**) Evolution of compression stress of CS LiOH-urea gel with different rinse time. (**c**,**d**) Relation between gelation behaviour and the concentration of LiOH (**c**) and the concentration of urea (**d**).

The thermal gelation process of CS solutions was studied by dynamic viscoelastic method (Fig. 4a and Supplementary Fig. 10). Storage modulus G' represented the elastic behaviour of the system, which was essentially related to the formation of junction points in the system. So the evolution of G' was followed with time when the system was cured at a predetermined temperature. Taking the G' curves of 40 °C as an example (Supplementary Fig. 10c). First, the heat absorption led to the rise of system temperature and consequently a minor decrease in G'. As system temperature continuously increased, a sharp increase in G' was observed. In this period, the storage modulus increased faster than loss modulus (G''), thus a crossover of G' and G'' curves occurred, which was usually determined as the gelation point^26^,^27^. In previous work, little attention was paid to the behaviour after this point. However, in fact G' continued increasing after the crossover, indicating that more junction points were formed. The increase of G' gradually slowed down. This was because the motion of CS chains was restricted, and the formation of more junction points became more difficult. Finally, the G' curve presented a plateau. In the thermal gelation stage, elevated temperature is the driving force of gelation. Different temperatures were used in the isothermal curing tests. When the predetermined temperature was 25 °C, the increase of G' could hardly be observed, while a minor increase was detected at 32 °C. The G' curve reached plateau more quickly when temperature was set at elevated value (60 and 80 °C). Although occurrence of plateau happened at different times, the evolution of G' curves presented similar pattern at all temperatures.

The plateau of G' indicated the end of thermal gelation. However, it did not mean the increase of toughness terminated. The variation of toughness was characterized by compression stress in the rinse stage. As shown in Fig. 4b, if not rinsed at all after thermal gelation, the CS gel proved to be brittle, which failed at a compression stress ≤0.05 MPa and strain ≤70%. During the rinse stage, the failure-compressive stress increased gradually. After LiOH and urea were completely removed from the system, CS hydrogel was formed. The hydrogel possessed failure-compressive stress 15 times higher than gel without rinsing, and reached a failure-compressive strain higher than 90%. This indicated that the system could absorb much more energy before fracture. The strength of CS hydrogel was mainly gained in the rinse stage.

To explore the driving force of strength increase, a serial of experiments were performed as control (Supplementary Fig. 9). The results showed that: (1) experimental groups with OH^−^, with or without urea, all proved to be brittle and had low failure strain; (2) experimental groups without OH^−^, even treated with urea or other inorganic salt (LiCl or NaCl), proved to be tough and had high failure-compressive strain. The results validated that, in the rinse stage, the removal of OH^−^ is the primary contribution to the increase of toughness.

The results above also indicated that LiOH and urea are not equal in the interaction with CS, so the further investigations were performed to clarify the different roles that LiOH and urea played during sol-gel transition (Fig. 4c,d and Supplementary Fig. 11). The occurrence of plateau was accelerated by the decrease of both the concentration of LiOH and urea, and was delayed contrariwise. However, the influence on the evolution of G' curves was quite different in the two cases. The *c*(LiOH) in the system determined the primary G' value (Fig. 4c). With lower *c*(LiOH), the primary G' value got closer to the value of plateau. However, the change of *c*(urea) did not affect the primary G' value nor the process of evolution (Fig. 4d). This also indicated that urea played a subordinate role of dissolution and gelation in the system.

**Figure 5:**
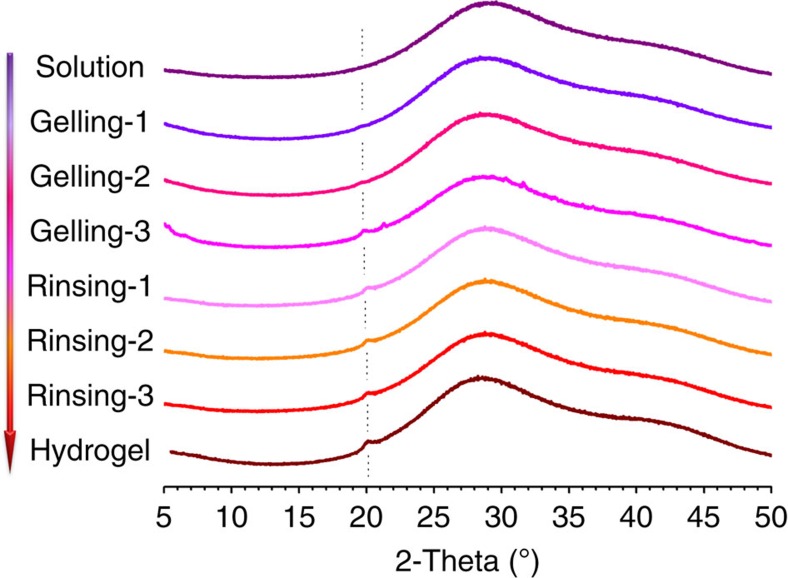
Evolution of crystalline during gelation process of CS LiOH-urea system. XRD patterns of CS LiOH-urea system in the entire gelation process.

Crystalline is a very important form of physical junction in hydrogel^28^. CS is a well-known semi-crystalline polymer in the solid state. So it is possible that the polymer chains would rearrange and form crystallites. The evolution of crystalline in the whole gelation process was studied by X-ray diffraction (Fig. 5). The broad peak belonged to the X-ray diffraction profile of liquid water (Supplementary Fig. 12). LiOH and urea in the solution did not show any peaks since they were at amorphous state. There was no characteristic peak of CS in the profile of CS LiOH-urea solution, this indicated that there was no crystalline in the solution before thermal gelation. With the initiation of thermal gelation, a small peak appeared at 2*θ*=20°. This peak could be attributed to the form II crystals of CS^29^. This demonstrated the formation of low amount of small crystalline. The crystalline was already formed in the elevated temperature and the amount of crystalline did not increase when the temperature decreased (Supplementary Fig. 13). Intensity of this peak increased with gelation time, indicating the formation of more crystalline. However, the intensity of peak did not increase in the rinse stage. The results indicated that the formation of crystalline mainly happened in the thermal gelation stage. The formation of crystalline was not affected by the introduction of tetraphenylethene (Supplementary Fig. 14).

## Discussion

We proposed the hypothesis on the evolution of CS LiOH-urea aqueous system during the gelation process.

**Figure 6:**
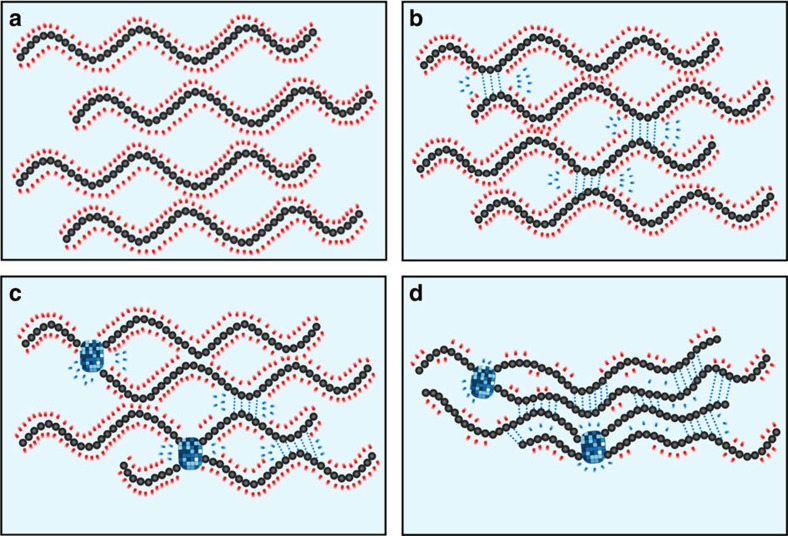
Schematic illustration of the formation of junction points of CS hydrogel in LiOH-urea solution. (**a**) Well-dissolved solution; (**b**) formation of inter/intramolecular hydrogen bonds of CS, induced by heating; (**c**) formation of crystalline in CS gel; (**d**) further formation of hydrogen bonds of CS, induced by the removal of LiOH and urea. The partial CS chains in the figure may belong to the same CS macromolecule or different CS macromolecules.

There existed dynamic intermolecular interactions among LiOH, urea and polysaccharide in the alkali/urea/cellulose solution^30^. On the molecular level, the oxygen atom in OH^−^ is electron-richer due to the extra electron, and consequently very competitive in the formation of hydrogen bonds with –NH_2_ and –OH on the CS chain. Several possible forms of hydrogen bonds between CS and OH^−^ were shown in Supplementary Fig. 15a. Due to the saturability of hydrogen bonds, these –NH_2_ and –OH can no longer form inter/intramolecular hydrogen bonds. When large number of –NH_2_ and –OH groups were attached by OH^−^, CS became soluble in this aqueous system (Fig. 6a). On the other hand, the role of urea in the intermolecular interaction is not decisive. If the OH^−^ detached from –NH_2_ and –OH on the CS chains, the oxygen atom in urea could serve as the hydrogen-bonding receptor for hydrogen atoms in these groups. Moreover, the hydrogen atoms in urea serve as the hydrogen-bonding donor for the electron-rich atoms in these groups (Supplementary Fig. 15b). Although the interactions are in a dynamic detached re-attached pattern, the addition of urea decelerates the formation of inter/intramolecular hydrogen bonds among CS.

The existence of terminal point in thermal gelation stage indicated the equilibrium of intermolecular interactions. The intermolecular interactions in the system can be described as the overall reaction of two competitive interactions. Reaction 1 is the formation of H-bonds between OH^−^ and CS, and reaction 2 is the formation of inter/intramolecular H-bonds among CS. The thermal gelation stage can be interpreted as the shift of equilibrium of the overall reaction at elevated temperature. The frequency of detach and re-attach of OH^−^ with CS increases with the rise of system temperature. Due to the restriction of macromolecule, reaction 2 is less susceptible to heat than reaction 1. So the equilibrium shifts to direction of forming more inter/intramolecular H-bonds among CS and even enable formation of crystalline (Fig. 6b,c). The termination of thermal gelation stage corresponds to the establishment of new equilibrium at the elevated temperature.

The rinse stage could also be interpreted with the shift of equilibrium. In this stage, the driving force is the concentration change of reactants. The removal of OH^−^ largely promotes the reverse course of reaction 1, and greatly promotes the formation of inter/intramolecular H-bonds among CS (Fig. 6d). So this stage is main stage for the increase of toughness and the volume shrinkage.

During the entire gelation process, the detachment of OH^−^ from CS chains render the possibility of crystallization. However, in the rinse stage, CS chains were not rearranged into the lattice. This could be explained that CS chains still had mobility in the early stage of gelation, and was able to reform and get into the lattice. While in the rinse stage, chains were bounded by inter/intramolecular hydrogen bonds formed in the thermal gelation. This indicated that the formation of H-bonds was more localized in the rinse stage.

The gelation process of CS LiOH-urea solution comprised two distinct stages. In the thermal gelation stage, the system showed similarity to typical thermogels (for example, PLGA-PEG-PLGA). However, there also exists difference in the gelation mechanism of the two-gelation system. The thermosensitivity of PLGA-PEG-PLGA originates from the balanced structure of hydrophilicity and hydrophobicity of the macromolecules^31^,^32^,^33^, while the thermosensitivity of CS LiOH-urea solution originates from the change of intermolecular interaction discussed above. The rinse stage can be considered as the removal of additives. The nature of this stage is transforming the medium to poor solvent by decreasing *c*(LiOH) and *c*(urea). Unlike random precipitation caused by introducing poor solvent, the structure of CS hydrogel further develops based on the embryonic structure formed in thermal gelation stage. Essentially, the two stages have something in common, which is the decrease of interactions between solvent and macromolecules and the increase of inter/intramolecular interactions of CS.

In this work, we visualized the entire gelation process of CS LiOH-urea aqueous system by AIE fluorescent imaging for the first time. Accompanied by other pseudo *in situ* investigations, we proposed the mechanism of gelation process. The formation of CS hydrogel via CS LiOH-urea aqueous system is unique. The entire process has two distinct but integrated stages, that is, the thermal gelation stage and rinse stage. Thermal gelation stage is driven by the elevated temperature, forming the embryonic structure and crystalline. While the rinse stage is driven by the continuous change of system components, greatly increasing interintramolecular hydrogen bonds. The two distinct stages can be interpreted under a unified understanding, which is the intermolecular interaction of solvent and polymer. The present work provided further understanding of the gelation process, and facilitated the utilization of polysaccharide. Furthermore, with the boom of AIE filed, this work could be a starting point, various AIE fluorogens would be available for the visualization of various gelation systems.

## Methods

### Materials and reagents

CS was prepared in our laboratory by heterogeneous N-deacetylation from commercial chitin. (Zhejiang Gold Shell Pharmaceutical Co., Ltd.) The average viscosity molecular weight (M*η*) of CS was 1.12 × 10^6^ Da and degree of deacetylation (DD) was 94.4%. M*η*, degree of deacetylation were determined following the process reported in ref. 34. Lithium hydroxide monohydrate (LiOH·H_2_O), urea and acetic acid were purchased from Sinopharm Chemical Reagent Co., Ltd. 1-[4-(isothiocyanatomethyl)phenyl]-1, 2, 2-triphenylethene was synthesized, and labelled onto CS chain to fabricate TPE-CS according to the procedures described in our previous work^21^.

### Preparation of TPE-CS solution and hydrogel

LiOH·H_2_O and urea were dissolved in deionized water to form a transparent solution with weight percentage of 4.8 and 8.0 wt.%, respectively. The resultant aqueous solution was used as solvent in this work. A certain amount of TPE-CS powder was mixed in the solvent. The mixture was treated by a freeze (−20 °C)-thawing (20 °C) process three times to get transparent TPE-CS LiOH-urea solution. For the study of solubility, 20 mg TPE-CS powder was mixed in 2 g solvent and then went through the dissolving procedure mentioned above. Fluorescence (FL) spectra of the TPE-CS LiOH-urea solution were measured on a Perkin-Elmer LS 55 spectrofluorometer. For the preparation of hydrogel, the concentration of TPE-CS was 2.0 wt.%. The solution was held for 30 min in a 60 °C thermostatic water bath, and turned to TPE-CS gel containing LiOH and urea. The gel was then fully washed in deionized water to remove LiOH and urea after the mould was unloaded.

### Fluorescent images

The fluorescent images of gelation process were obtained by confocal laser scanning fluorescence microscope (Leica TCS SP5 CLSM). A TPE-CS LiOH-urea aqueous solution was prepared first, and the concentration of TPE-CS was 2 wt.%. The solution was then filled in a quartz culture dish and the original temperature of solution was −4 °C. In thermal gelation stage, the heat was provided by a heat source with constant temperature (60 °C). The timing started once the sample contacted with the heat source. The structure of TPE-CS hydrogel was observed after the LiOH and urea was completely removed.

### Transparency

The light transmission of the CS solution, gel and hydrogel was examined at room temperature using a WGT-S Haze and Luminous Transmittance Instrument. The solution was first filled in a cuvette with thickness of 10 mm and side length of 40 mm. The predetermined temperature of heat source was 60 °C in the thermal gelation stage. In the rinse stage, the CS gel was unloaded from the cuvette and supported by a sample holder during tests. The gel sample was rinsed in 500 ml deionized water before every test.

### Shrinking behaviours of the hydrogel

The test of volume shrinking behaviours of CS LiOH-urea system were carried out by measuring the diameter change of hydrogel discs^35^. The mould was unloaded after enough thermal gelation time, and then the gel was rinsed for 5 min in 100 ml deionized water before every measurement.

### Rheological tests

Rheological tests were performed on AR-G2 rheometer (TA Co., USA). The freshly prepared and degassed CS solution were used as samples, with *c*(CS)=2 wt.%. Silicon oil was placed around the rim to prevent water evaporation during the measurement. The temperature was set to 4 °C first to prevent gelation of sample, and then maintained at a predetermined temperature as soon as the test started. The change in dynamic modulus was monitored as a function of time during the gelation process. All the rheological experiments were performed within the linear viscoelastic region.

### Mechanical tests

The mechanical property of CS hydrogels were obtained on a universal materials testing machine (Instron, 5543A) at a strain rate of 2% min^−1^ for compression tests at room temperature. Samples were prepared to be cylinder. The mould used in this section had a diameter of 33 mm and a depth of 4 mm. Several identical samples were prepared and went through thermal gelation stage. Then the samples were rinsed in 200 ml deionized water for desired time, respectively.

### X-ray measurements

Wide-angle X-ray diffraction profiles were collected with an Ultima IV analytical instrumentation (Rigaku Corporation) using Ni-filtered Cu Kα radiation scanned at 2 deg (2*θ*) s^−1^ in the 2*θ* range 5–60°. The solution was first filled in a tailor-made specimen holder with thickness of 2 mm. The predetermined temperature of heat source was 60 °C in the thermal gelation stage. The sample was heated by the source for 5 min before every measurement. The treatments were denoted as gelling 1, gelling 2 and gelling 3. After thermal gelation the gel was rinsed for 30 min in 500 ml deionized water before every measurement. The treatments were denoted as rinsing 1, rinsing 2 and rinsing 3.

### Data availability

The data that support the findings of this study are available from the corresponding author on request.

## Additional information

**How to cite this article:** Wang, Z. *et al*. Gelation process visualized by aggregation-induced emission fluorogens. *Nat. Commun.* 7:12033 doi: 10.1038/ncomms12033 (2016).

## Supplementary Material

Supplementary Information Supplementary Figures 1-15 and Supplementary Discussion
