# Supplementary material for: Gelation process visualized by aggregation-induced emission fluorogens
Source: Nat Commun. 2016 Jun 23;7:12033. doi: 10.1038/ncomms12033 (PMC4931011; doi:10.1038/ncomms12033)
Supplement: Supplementary Information — Supplementary Figures 1-15 and Supplementary Discussion [file ncomms12033-s1.pdf]

Supplementary Figure

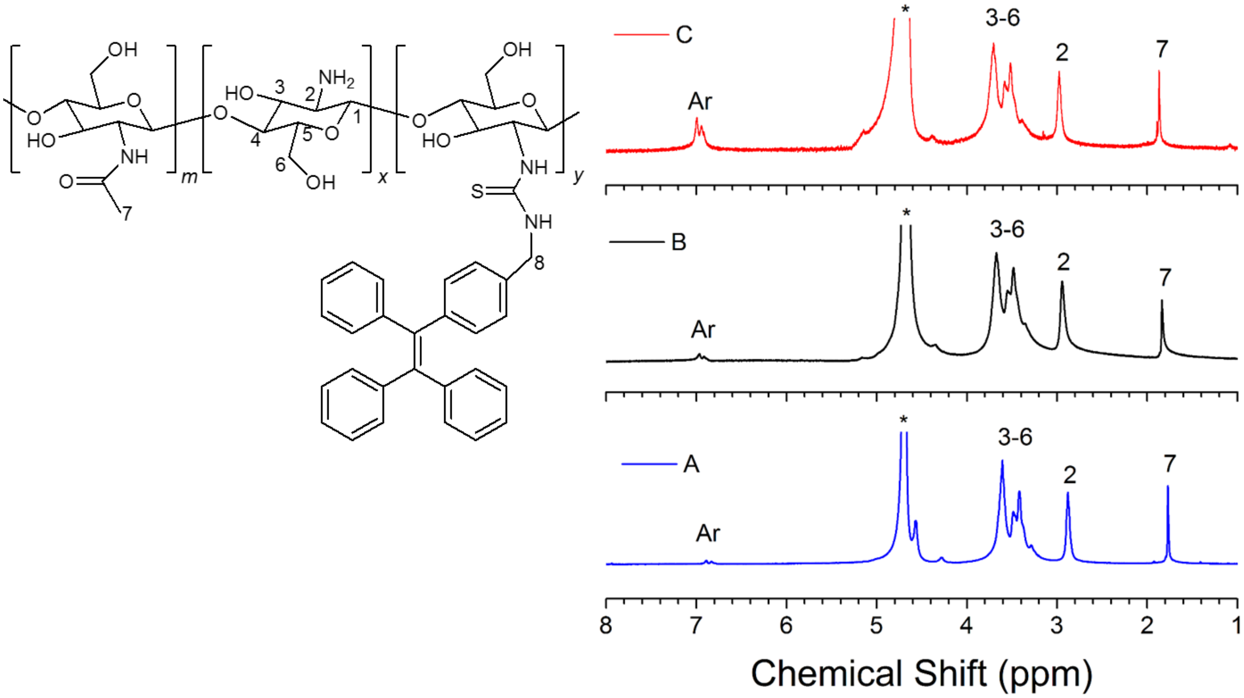

Supplementary Figure 1 | ^{1}H NMR spectra of TPE-CS. Sample A, B, and C measured in a trifluoroacetic acid-d/water-d_2 mixture at room temperature. The solvent peaks are marked with asterisks.

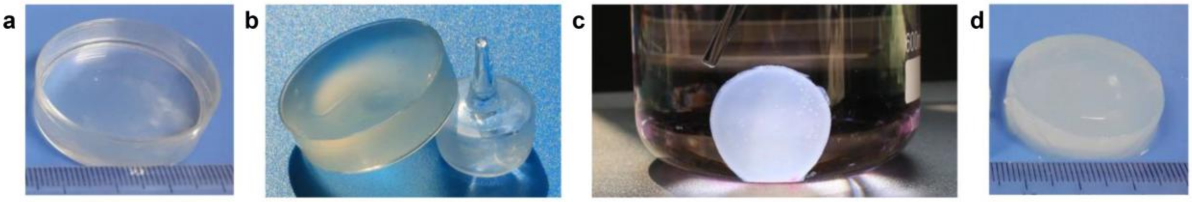

Supplementary Figure 2 | Typical preparation procedures of CS hydrogel via LiOH-urea solvent. a, CS LiOH-urea aqueous solution; **b,** CS gel formed after thermal gelation, containing LiOH and urea; **c,** rinse procedure of CS gel; **d,** CS hydrogel, with LiOH and urea completely removed.

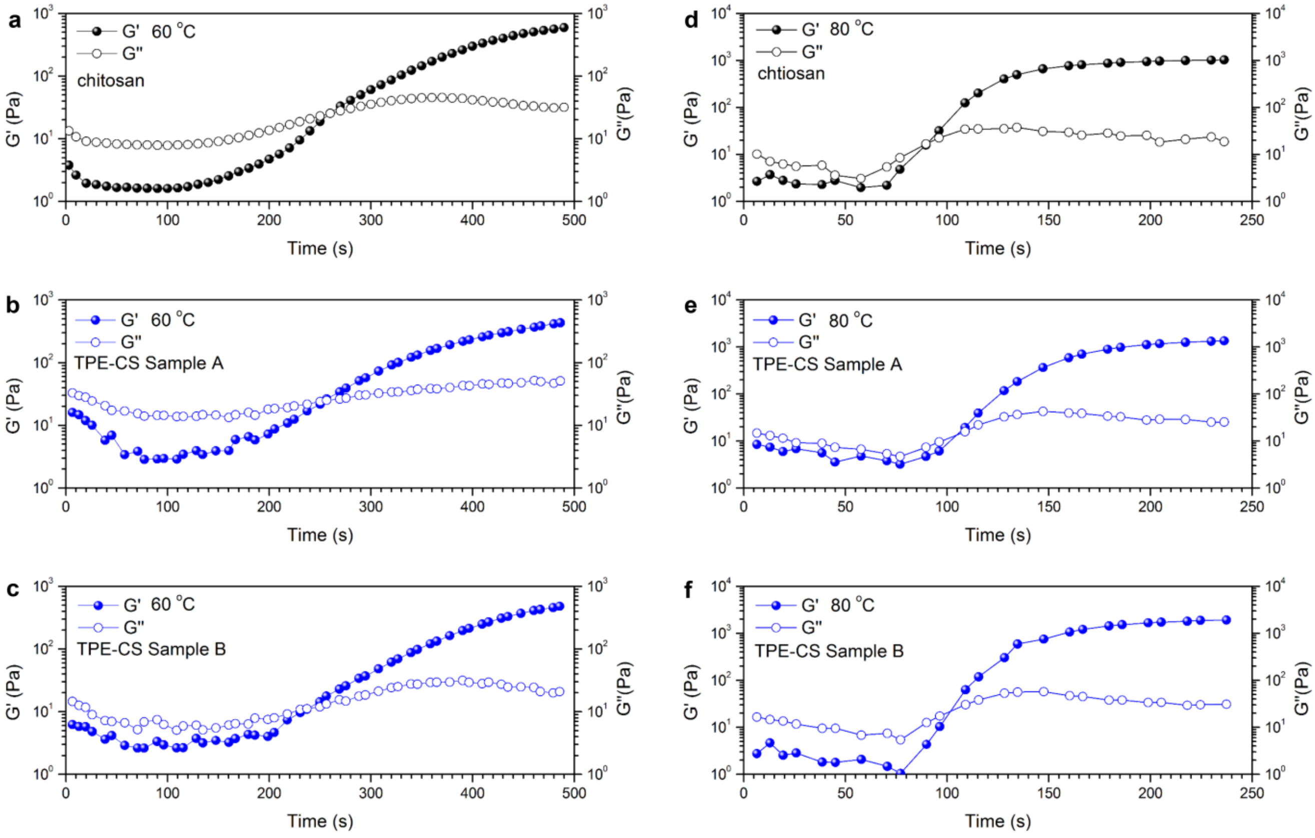

Supplementary Figure 3 | Dynamic modulus of 2 *wt.*% CS or TPE-CS LiOH-urea solution. a, d, CS sample; **b, e,** TPE-CS Sample A**; c, f,** TPE-CS Sample B.

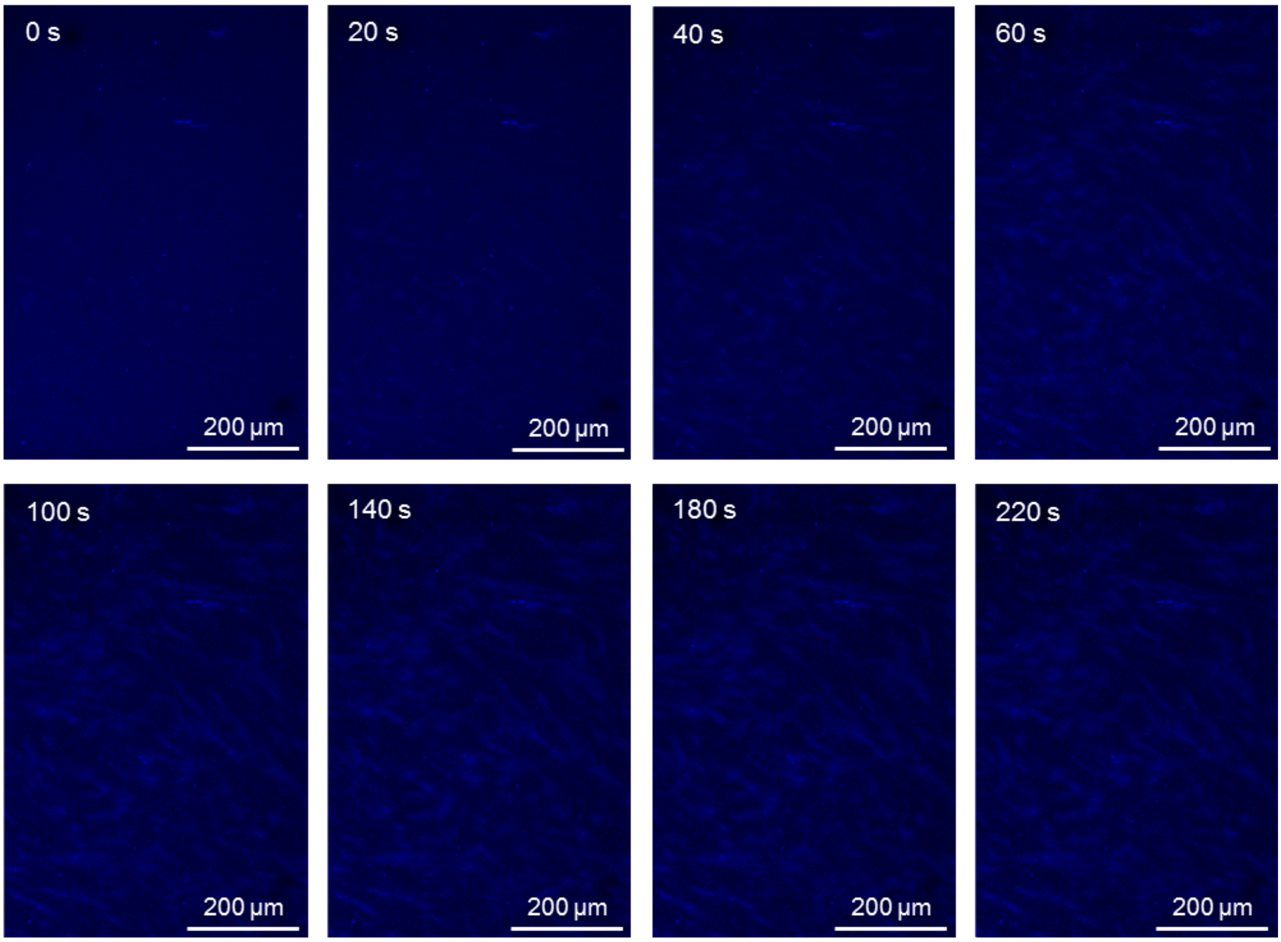

Supplementary Figure 4 | Structural Evolution during thermal gelation stage. Confocal laser scanning fluorescence microscope images of the thermal gelation process of TPE-CS.

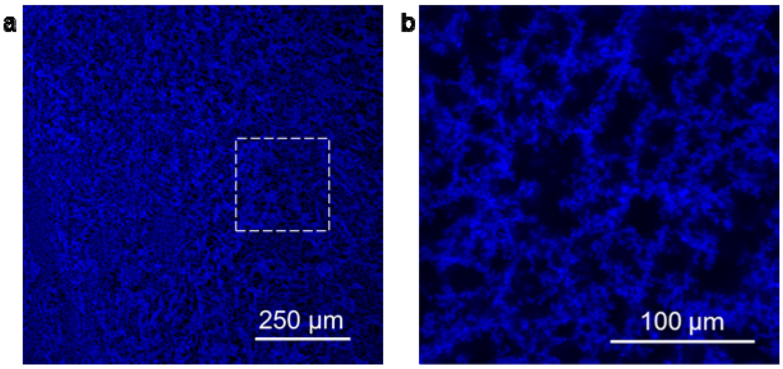

Supplementary Figure 5 | Confocal laser scanning fluorescence microscope images of the hydrogel. Images were obtained at the surface of the sample only to give a better vision of the structural details.

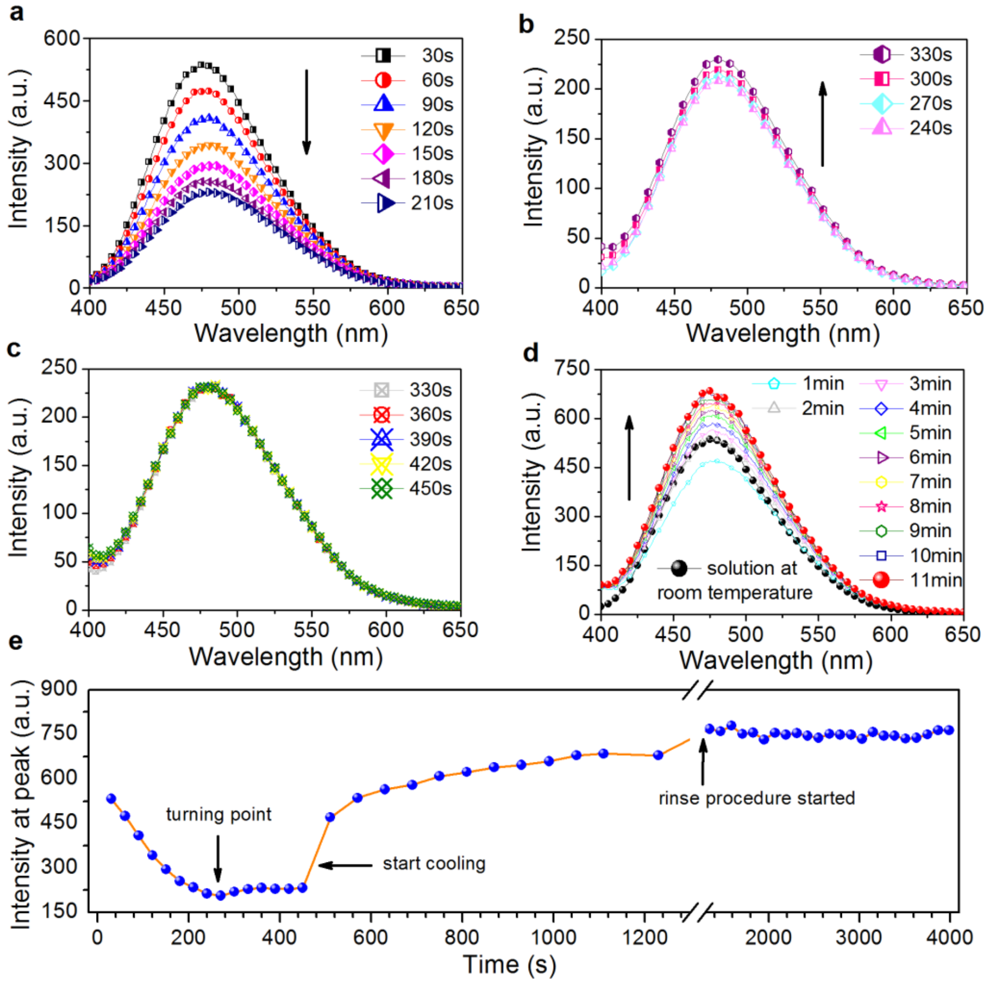

Supplementary Figure 6 | Fluorescence response of TPE-CS (sample B) LiOH-urea solution during the gelation process. a-d, Emission spectra of TPE-CS system in the gelation process: heating process **(a-c)** and cooling process **(d)**. The sample was tested with a time interval of 30 s in the thermal gelation stage **(a-c)**; **e,** Relationship between the fluorescence intensity at the emission peak 479 nm of TPE-CS and time in the entire gelation process.

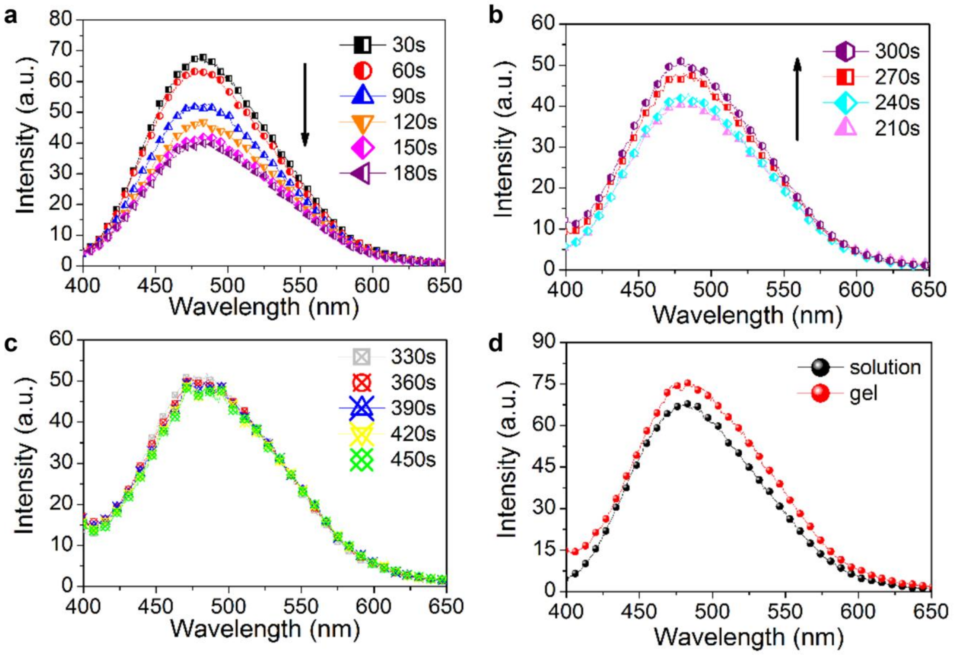

Supplementary Figure 7 | Fluorescence response of TPE-CS (sample A) LiOH-urea solution during the gelation process. **a-c,** heating process, the sample was tested with a time interval of 30 s; **d,** gel after cooling process**.**

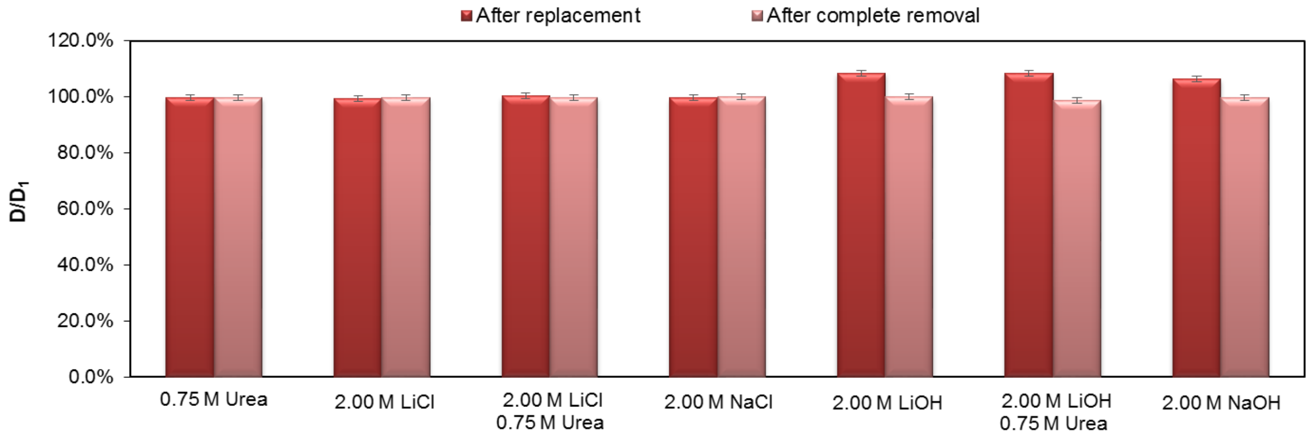

Supplementary Figure 8 | Dimension shrinkage rate of CS gel. D_1 the diameter of the CS hydrogel. Water held within the hydrogel was completely replaced by certain aqueous solution, then the hydrogel samples were rinsed with deionized water to remove the solution components.

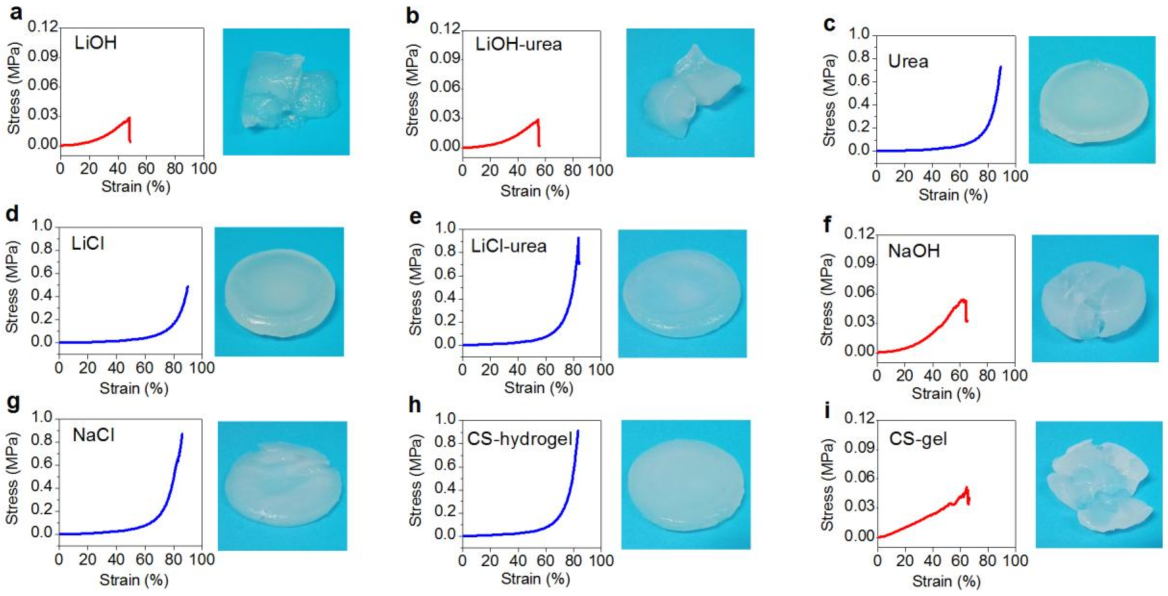

Supplementary Figure 9 | Compression stress of CS gel samples containing different aqueous solutions. The scales of Y-axis were not identical in all sub-figures, mold used in this section had a diameter of 33 mm and a depth of 7 mm.

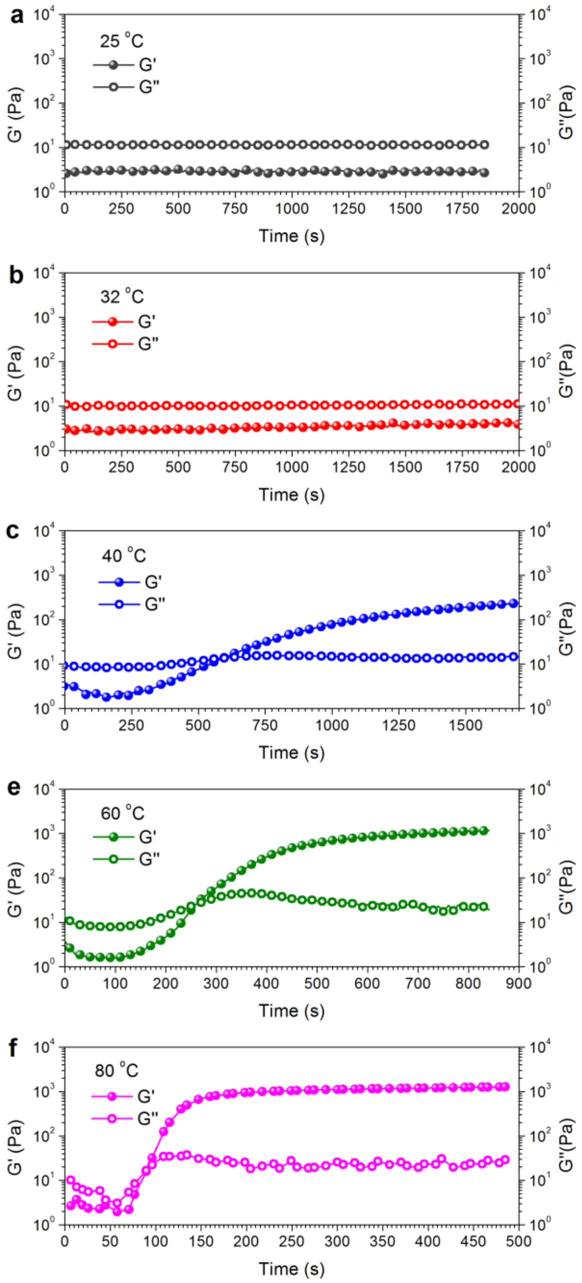

Supplementary Figure 10 | Evolution of dynamic modulus of CS LiOH-urea solution with time at different temperature. a, 25 ºC; **b,** 32 ºC; **c,** 40 ºC; **d,** 60 ºC; **e,** 80 ºC.

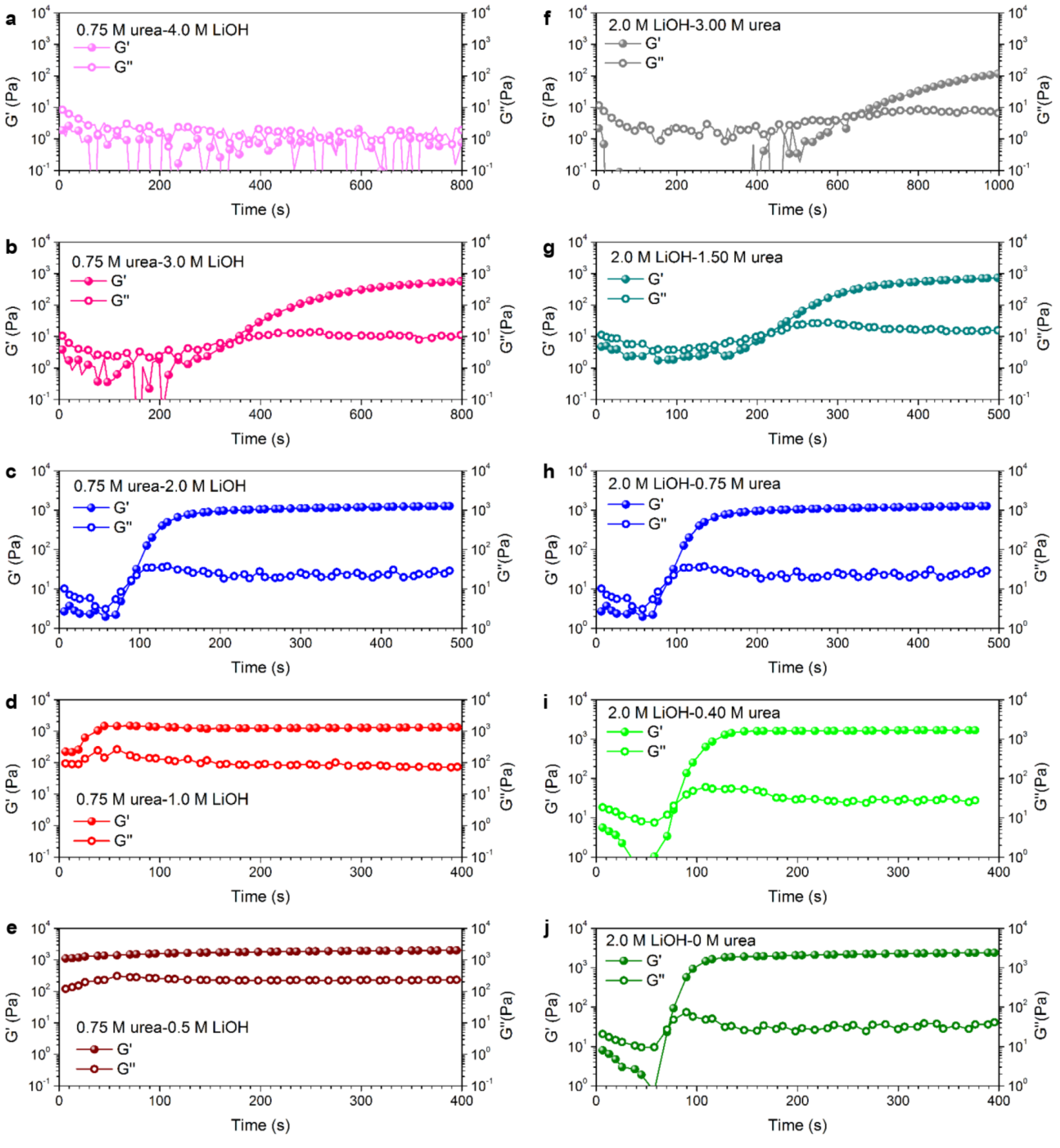

Supplementary Figure 11 | Evolution of dynamic modulus of CS LiOH-urea solution with different concentration of LiOH or different concentration of urea at 80 °C. a, 0.75 M urea-4.0 M LiOH; **b,** 0.75 M urea-3.0 M LiOH; **c,** 0.75 M urea-2.0 M LiOH; **d,** 0.75 M urea-1.0 M LiOH; **e,** 0.75 M urea-0.5 M LiOH. **f,** 2.0 M LiOH-3.00 M urea; **g,** 2.0 M LiOH-1.50 M urea; **h,** 2.0 M LiOH-0.75 M urea; **i,** 2.0 M LiOH-0.40 M urea; **j,** 2.0 M LiOH-0 M urea.

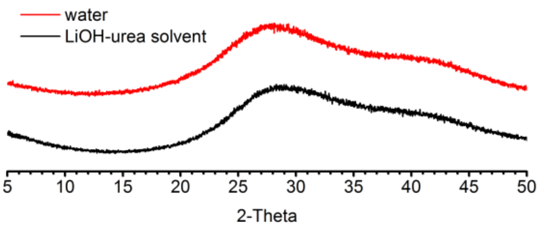

Supplementary Figure 12 | XRD profile of aqueous environment of the CS LiOH-urea system. XRD patterns of water and LiOH-urea solvent, respectively.

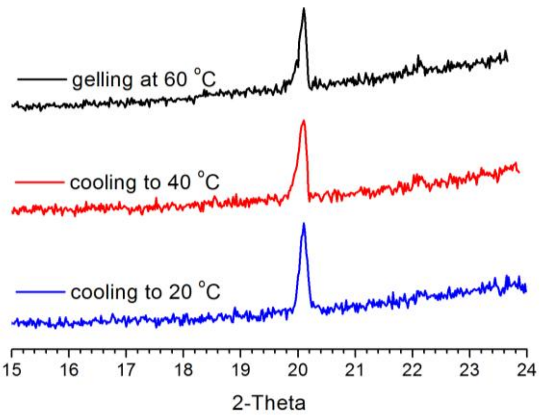

Supplementary Figure 13 | Evolution of crystalline in cooling process. XRD patterns of CS LiOH-urea system tested under different temperature conditions.

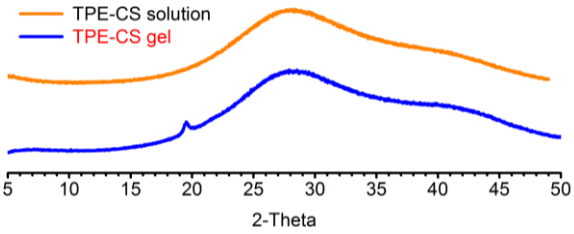

Supplementary Figure 14 | The influence of TPE fluorogens on the formation of crystalline. XRD patterns of TPE-CS LiOH-urea solution and gel *in-situ* formed.

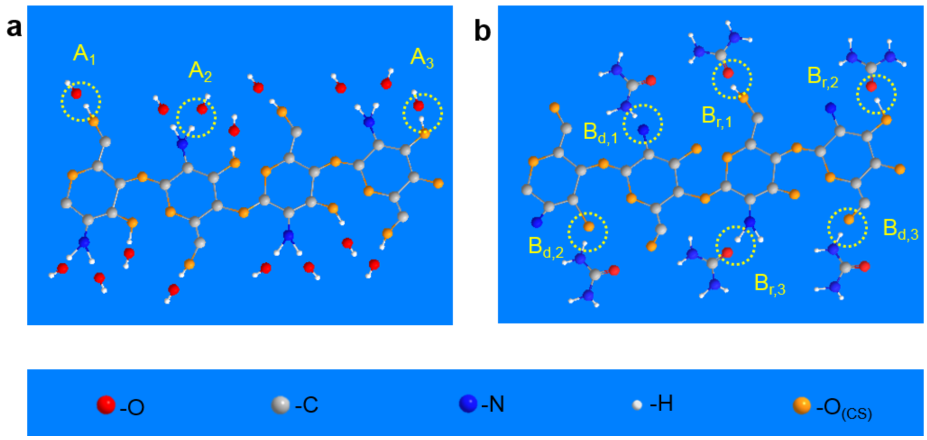

Supplementary Figure 15 | Schematic representation of possible intermolecular interactions in CS LiOH-urea solution. a, Possible interaction between OH^- and CS, A_1、A_2、A_3 represented the hydrogel bonds formed by $OH^-$and C_6-OH、-NH_2、C_3-OH, respectively. **b,** Possible interaction between urea and CS, $B_{r,1}$, $B_{r,2}$, $B_{r,3}$ represented that the oxygen atom in urea could serve as the hydrogen-bonding receptor for hydrogen atoms in–NH_2 and –OH on the CS chains; $B_{d,1}$, $B_{d,2}$, $B_{d,3}$ represented that the hydrogen atoms in urea serve as the hydrogen-bonding donor for the electron-rich atoms in these groups. H atoms in CS macromolecules were omitted except those interacted with OH^- or urea.

Supplementary Discussion

In thermal gelation, the FL intensity went through a decrease-increase-equilibrium process. This was due to the counter-balance of two factors: the system temperature and the degree of aggregation. The FL intensity was reduced by temperature rise owing to alleviation of restriction of intramolecular rotation (RIR), and was promoted owing to the strengthening of RIR. The decline of FL intensity corresponded to the heat absorption of system (Supplementary Fig. 6a). Then with the rise of system temperature, the sol-gel transition initiated. The formation of junction points lead to cross-linked macromolecular chains. The TPE attached to the CS backbones got wrapped in the cross-linked chains, thus promoted the RIR. So the FL intensity increased and gradually became stable (Supplementary Fig. 6b, c), which was in accordance with the fluorescent images. The FL intensity recovered as the temperature dropped in the cooling process (Supplementary Fig. 6d). Due to the AIE behaviour and the aggregation state of system, the gel possessed higher FL intensity than solution under the same system temperature. In the rinse state, the fluorescence intensity remained unchanged. This was because that after thermal gelation, the phenyl rotors of TPE were already strangled by the cross-linked CS chains. The same tendency can be observed with Sample A (Supplementary Fig. 7). This indicated that, with low degree of labelling, the introduction of TPE fluorogens did not exert much interference on the gelation process of CS.

Change of gel volume and toughness indicated that ((Supplementary Fig. 8), for solutions without OH^-, the volume of CS hydrogel barely changed after replacement of aqueous solution held within the gel. The volume barely changed after the components were completely removed neither. However, the volume of CS gel showed evident change when the aqueous solution contained OH^-. Thus the removal of OH^- is closely related to the volume change of CS gel.

When the temperature was low (Supplementary Fig. 10a), G' and G'' showed no increasing tendency in a long time range, and there was no crossover of G' and G'' curves. When the temperature increased, although no crossover was observed within a relatively long time, the G' curve showed an increasing tendency (Supplementary Fig. 10b). When the temperature further increased, the crossover of G' and G'' curves occurred, and the crossover time decreased with the increase of temperature (Supplementary Fig. 10c-e). As demonstrated in the main text, G' continued to increase after the crossover, indicating that more junction points were formed.

When the *c*(LiOH) was high (Supplementary Fig. 11a), gelation of solution did not happen in a long time range. With the decrease of *c*(LiOH), the G' and G" showed an increasing tendency, and the crossover of G' and G" curves occurred (Supplementary Fig. 11b, c). When *c*(LiOH) further decreased (Supplementary Fig. 11d, e), the G' already dominated G". However, the CS LiOH-urea sample was solution with very high viscosity rather than a gel with particular shape. This indicated that the lower the *c*(LiOH) was, the closer the system was to the equilibrium of thermal gelation. Although the gelation process was accelerated by the decrease of *c*(urea), the influence of *c*(urea) was quite different from that of *c*(LiOH). With the decrease of *c*(urea), the crossover time decreased. But the decrease of c(urea) did not change the primary dynamic modulus of solution, nor the pattern of evolution. This also indicated that urea played a subordinate role of dissolution and gelation in the system.
